# Ethanol and C2 ceramide activate fatty acid oxidation in human hepatoma cells

**DOI:** 10.1038/s41598-018-31025-0

**Published:** 2018-08-27

**Authors:** Jason M. Correnti, Lauren Gottshall, Annie Lin, Bianca Williams, Amanke Oranu, James Beck, Jie Chen, Michael J. Bennett, Rotonya M. Carr

**Affiliations:** 1 Department of Gastroenterology, University of Pennsylvania, Perelman School of Medicine, Philadelphia, PA 19104 USA; 2 Palmieri Metabolic Laboratory at Children’s Hospital of Philadelphia, Philadelphia, PA 19104 USA

## Abstract

Obesogenic lipids and the sphingolipid ceramide have been implicated as potential cofactors in alcoholic liver disease (ALD) patients. However, the mechanisms by which these lipids modulate lipid trafficking in ethanol-treated human liver cells to promote steatosis, an early stage of ALD, are poorly understood. We measured fatty acid (FA) uptake, triglyceride export, FA synthesis and FA oxidation in human hepatoma (VL-17A) cells in response to ethanol and the exogenous lipids oleate, palmitate and C2 ceramide. We found that in combination with ethanol, both oleate and palmitate promote lipid droplet accumulation while C2 ceramide inhibits lipid droplet accumulation by enhancing FA oxidation. Further, using both a pharmacologic and siRNA approach to reduce peroxisome proliferator-activated receptors α (PPARα) gene expression, we demonstrate that C2 ceramide abrogates ethanol-mediated suppression of FA oxidation through an indirect PPARα mechanism. Together, these data suggest that lipids interact differentially with ethanol to modulate hepatocellular lipid droplet accumulation and may provide novel targets for preventing the earliest stage of alcoholic liver disease, alcoholic steatosis.

## Introduction

Chronic ethanol consumption can cause alcoholic steatosis, the excessive accumulation of lipids within hepatocellular cytoplasmic lipid droplets (LDs). Alcoholic steatosis subsequently predisposes patients to the clinical spectrum of alcoholic liver disease (ALD) including steatohepatitis, fibrosis and cirrhosis^1^. Consequently, perturbation of hepatocyte lipid trafficking by ethanol is important for both ALD inception and progression.

Hepatocellular LDs are comprised primarily of a core of neutral triglycerides (TGs) surrounded by a phospholipid monolayer of associated LD proteins. Chronic ethanol intake has been demonstrated to promote TG accumulation within these LDs by enhancing fatty acid (FA) uptake from the circulation^2^, reducing export of the TG-rich very low density lipoprotein (VLDL) particle^3^, upregulating pathways involved in *de novo* lipogenesis (DNL)^2,4–6^, inhibiting FA β-oxidation^6^, and upregulating the major hepatic LD protein Perilipin 2 (PLIN2)^7,8^, a protein we previously demonstrated is required for the development of alcoholic steatosis in mice^7^.

In addition to the independent effects of ethanol on LD accumulation, the combined steatogenic effect of excessive ethanol intake and overnutrition is being increasingly recognized^9–11^, suggesting that ethanol’s effects on hepatic lipid metabolism can be modulated by nutritional status. For example, several studies suggest that saturated FAs protect against while polyunsaturated FAs promote experimental ALD in rodents^12–14^. You *et al*. showed that mice fed a diet containing ethanol equivalent to 27.5% of calories for four weeks with fat calories derived from polyunsaturated corn oil had significantly more liver damage than mice fed a diet supplemented with saturated fat derived from cocoa butter^14^. While a positive correlation between dietary linoleic acid content and liver damage in alcohol fed rats has been demonstrated^13^, there is little molecular detail on how specific FAs alter lipid trafficking. Further, it is unknown whether other lipid species similarly interact with ethanol to either promote or ameliorate alcoholic steatosis.

Ceramides are bioactive sphingolipids that have previously been implicated in the pathogenesis of ALD^7,15–18^. Ceramides are comprised of a sphingosine backbone and hydrophobic tail of FAs of varying lengths. Long-chain ceramides play a significant role in signal transduction, apoptosis^19^, inhibition of insulin signaling^9,20^ and AMP-activated Protein Kinase (AMPK) activation^21^. We and others have demonstrated that hepatic long-chain ceramide content increases in humans^22^ and rodent models of alcoholic steatosis^7,21,23–25^. Pharmacologic reduction of liver ceramides is associated with decreased hepatic steatosis in ethanol-fed mice^15,17,26^. Although short-chain ceramides have not similarly been associated with ALD pathogenesis, short-chain ceramides such as C2 have been used experimentally because they are cell permeable^27^ and mimic the physiological effects of long-chain ceramides, such as induction of apoptosis^28^ and inhibition of insulin signaling^28,29^. This may be due in part to conversion of short-chain ceramides to long chain ceramides through their re-acylation to form long-chain ceramides^30^. Short-chain ceramides alter lipid trafficking by reducing cell surface expression of the FA transporter, Cluster of Differentiation 36 (CD36), in macrophages^31^ and by altering VLDL secretion in hepatoma cells^32^. However, the mechanism by which ceramides specifically modulate ethanol’s effects on hepatic lipid metabolism is unknown.

Progress in understanding the potential interactions between ethanol and exogenous lipids in hepatocellular lipid biology has been hindered in part by a lack of tractable *in vitro* systems. In the liver, ethanol is oxidized to acetaldehyde by alcohol dehydrogenase (ADH) and cytochrome P450 isoform 2E1 (Cyp2E1) and to acetate by acetaldehyde dehydrogenase. Many hepatoma cell lines, including HepG2 cells, lack alcohol-oxidizing enzymes^33^ and *in vitro* cultured primary hepatocytes rapidly lose ethanol-oxidizing capacity^34,35^. VL-17A cells are derived from HepG2 cells and are stably transfected with murine ADH and human Cyp2E1^36^. These cells are of human origin, have been used to gain insight into human hepatocellular metabolism^37,38^, and have been demonstrated to accumulate LDs in response to ethanol^26^ and high dose lipotoxic stimuli^39^.

We used VL-17A cells to develop an *in vitro* model of alcohol- and exogenous lipid-induced hepatic steatosis and performed a systematic analysis of lipid handling in cells co-incubated with ethanol and the unsaturated FA oleate, saturated FA palmitate and C2 ceramide. We measured effects on FA uptake, synthesis, oxidation and TG export and sought to investigate the relative contribution of these pathways to hepatocellular LD accumulation and PLIN2 regulation. Herein, we demonstrate that ethanol increases TG levels primarily through inhibition of FA oxidation and that exogenous lipids have species-specific effects on FA oxidation, leading to differential effects on cellular TG levels in the presence of ethanol. We also report for the first time a protective effect of C2 ceramide on ethanol-mediated LD accumulation.

## Materials and Methods

### Cell culture

VL-17A cells were a generous gift of Dr. Dahn Clemens, University of Nebraska. Cells were maintained at 37 °C with 5% CO_2_ in Dulbecco’s Modified Eagle’s Medium (GE Healthcare Hyclone, Little Chalfont, UK) supplemented with 10% fetal bovine serum, penicillin (100 units/ml) and streptomycin (100 µg/ml). 1 × 10^6^ cells were plated for RNA isolation, intracellular TG and non-esterified FA (NEFA) measurements. 3 × 10^6^ cells were plated for all other assays. Cells were incubated with standard media; or supplemented with 100 mM ethanol and C2 ceramide (10 µM) (Cayman Chemical, Ann Arbor, MI), oleate (100 µM) (Sigma, St. Louis, MO) or palmitate (40 µM) (Sigma). Oleate and palmitate were complexed to 5% FA free bovine serum albumin (BSA, Gemini Bioproducts, West Sacramento, CA) prior to addition to the media. Cells were given fresh media at 24 h intervals. Cell viability was assessed by CellTiter 96® Aqueous One Solution Cell Proliferation Assay (Promega, Madison, WI), according to manufacturer’s instructions.

### Transfection

For peroxisome proliferator-activated receptor (PPAR) α knockdown experiments, cells were transfected with 10 nM PPARα siRNA (Santa Cruz Technology, Santa Cruz, CA) or silencer select control siRNA (Thermo Fisher Scientific, Waltham, MA) using RNAiMax lipofectamine reagent (Thermo Fisher Scientific). Cells were assayed for knockdown 6 days following transfection. For PPARα reporter assays, cells were transfected with Cignal PPAR Reporters (Qiagen, Germantown, MD) using Lipofectamine 2000 (ThermoFisher Scientific) and cells were assayed 48 h later.

### FA oxidation

Six hours prior to harvest, cells were washed with 1× PBS (Hyclone) and assay buffer was added. Assay buffer contains 325 mM oleate, 33 nM ^3^H-oleate (American Radiochemicals, St. Louis, MO) conjugated to FA free BSA and dissolved in Krebs-Henseleit Buffer (118 mM sodium chloride, 4.7 mM potassium chloride, 1.2 mM magnesium sulfate, 1.25 mM calcium chloride 1.2 mM potassium phosphate, 25 mM sodium bicarbonate and 11 mM glucose). Following incubation for 6 h at 37 °C without CO_2_, cell supernatants were collected for ^3^H_2_O quantitation, and remaining cells were lysed for protein quantitation by BCA assay. Cell supernatants were precipitated by mixing 1:1 with 10% trichloroacetic acid and neutralized by addition of sodium hydroxide to a final concentration of 0.73 M. Neutralized supernatants were run over columns packed with AG 1-X8 Resin (Biorad, Hercules, CA). Flow through was added to scintillation fluid, incubated overnight at ambient temperature and counted on a LS6500 scintillation counter (Beckman Coulter, Brea, CA). Samples were normalized to cell protein content.

### Acetate incorporation assay

100 µM ^3^H-acetate (Perkin Elmer, Waltham, MA) was added to cells two hours prior to harvest. Cells were washed twice in 1× PBS and were lysed in 5% triton X-100. Following protein determination, cell lysates were extracted with chloroform/methanol (2:1) followed by addition of magnesium chloride to a final concentration of 1.64 mM. Following vortexing and centrifugation, the organic phase was extracted three times and dried at 70 °C, resuspended in 100% ethanol, and counted in scintillation fluid.

### Mitochondrial stress tests

Cells were plated in Xfe96 culture plates at a density of 1 × 10^5^ cells per well and allowed to attach overnight. Cells were treated for 48 h with 100 mM ethanol, with and without 10 µM methylene blue (MB) treatment for 24 h. One hour before assay, cells were transferred into Seahorse Assay Media with 2 mM GlutaMAx (Agilent, Santa Clara, CA) supplemented with 5 mM glucose with ethanol and MB as needed to maintain treatment continuity. Cells were then placed at 37 °C for one hour without CO_2_. After pre-incubation, oxygen consumption rates were measured using a Seahorse XFe96. Mitochondrial function was assessed during stress tests by sequential addition of oligomycin (1 µM), carbonyl cyanide-4-(trifluoromethoxy)phenylhydrazone (FCCP, 1 µM), and a mix of rotenone and antimycin A (0.5 µM).

### Cell staining and Oil Red O quantification

Cells were incubated on plates coated with 3 µg/cm^2^ poly-D-lysine (EMD Millipore, Billerica, MA), fixed in 10% formalin (Sigma). For Nile red staining, fixed cells were washed three times in 1× PBS, and treated for 10 minutes at 37 °C with a 1 µM Nile red solution prepared in DMSO. Cells were then washed once with 1× PBS, counterstained with DAPI (4′,6-diamidino-2-phenylindole) and washed again with water. For Oil Red O staining, fixed cells were treated with a filtered 0.5% Oil Red O (Sigma) solution prepared in isopropanol. Cells were then washed with deionized water and pictures were taken using Nikon Eclipse Ti-U confocal microscope (Nikon, Melville, NY) fitted with a QiCam (Qimaging, Surrey, BC). LDs were quantified by manual counting using ImageJ software^40^. For Oil Red O quantitation, stained cells were dried and Oil Red O was eluted in isopropanol with gentle shaking. Eluted Oil Red O was quantified in supernatants by spectrophotometry at 500 nM.

### PPARα DNA-binding Assay

VL-17A nuclei were purified using the Nuclear Extraction Kit (Origene, Rockville, MD) and nuclear protein was quantified by BCA assay. PPARα DNA-binding in 7 µg of nuclear protein was measured using the Transcription Factor Assay Kit (Cayman Chemical) according to manufacturer’s instructions.

### AMPK activity assays

Cells were washed twice with 1× PBS and lysed in 50 mM Tris, 250 mM mannitol, 1 mM EDTA, 1 mM EGTA, 1 mM DTT, 1% triton X-100, 50 mM sodium fluoride, 5 mM sodium pyrophosphate plus protease inhibitor tablet (Roche, 1 tablet/10 ml). Protein content was normalized by BCA assay. An equal amount of protein was added to AMPK activity assay buffer containing 62.5 mM HEPES, 62.5 mM NaCl, 1.25 mM EDTA, 1.25 mM EGTA, 1 mM DTT, 5 mM MgCl_2_, 1 mM adenosine monophosphate, 2.5 mM adenosine triphosphate, 50 µCi ATP Gamma 32 P (10 mCi/ml, Perkin Elmer), 6.25 mM sodium pyrophosphate plus protease inhibitor tablet. Samples were incubated with and without 1 mM SAMS peptide (Abcam) for 10 min at 30 °C. Reactions were spotted on P81 Whatman paper square (EMD Millipore) and air dried. Squares were washed three times for five minutes with 1% phosphoric acid, once for five minutes in water, and once in acetone for five minutes. Squares were then air dried, added to scintillation fluid and counted as described above. Radioactive counts in samples without SAMS peptide was considered background.

### Lipid analysis

Cell supernatants were collected prior to harvest for quantitation of extracellular NEFA and TG. For assessment of intracellular TG and NEFA, cells were lysed in 5% triton X-100 followed by three cycles of a five minute incubation at 80 °C, vortex and five minutes cool to room temperature. Colorimetric enzymatic assays were used to measure NEFA (Wako Diagnostics, Richmond, VA) and TG (Stanbio, Boerne, TX).

### Lipid droplet isolation

Cells were collected following trypsin treatment, washed three times in 1× PBS and resuspended in a pH 6.8 solution containing 100 mM K2HPO4/KH2PO4, 5 mM MgCl_2_, proteinase and phosphatase inhibitors. Cells were lysed with a dounce homogenizer and centrifuged to remove cell debris. LDs were purified on a gradient of 1.3, 0.86 and 0.25 M sucrose, with the cell lysates placed between the 0.86 and 0.25 M layers. Following centrifugation at 100,000xg for 1 h, the LD fraction was collected and washed three times with 0.25 M sucrose.

### Quantitative real-time RT-PCR

RNA was extracted using Purelink RNA mini kit (ThermoFisher Scientific) with on-column DNaseI treatment (ThermoFisher Scientific) and quantitated by nanodrop. 2 µg of RNA was reverse transcribed using the High Capacity cDNA Reverse Transcription Kit (ThermoFisher). mRNA was quantified by real-time RT-PCR using Taqman Gene expression master mix (ThermoFisher) and Taqman primers (ThermoFisher) run on the SteponePlus Real-Time PCR machine (ThermoFisher). mRNA expression was normalized to 18s ribosomal RNA.

### Western blotting

Cells were lysed in protein lysis buffer (1% NP-40, 0.5% Triton, 10% glycerol, 150 mM NaCl, 1 mM EDTA, 0.5 M Tris) with complete protease and phosphatase inhibition cocktail tablets (Roche, Penzberg, Germany) and protein content was measured by BCA asay (ThermoFisher Scientific). 20 µg of protein were separated by 4–12% Nupage Bis-Tris gel (ThermoFisher) and wet transferred on ice to nitrocellulose membranes for 1 hr at 100 Volts. In some cases, the gels were cut and strips from multiple gels were transferred together^41^. Membranes were blocked with 5% milk in TBST and incubated overnight at 4 °C with antibodies recognizing PLIN2 (1:1000, Abcam, Cambridge, MA)^7^, GAPDH (1:1000, Millipore)^42^, phosphorylated or total AMPK^43,44^ and ACC^45,46^ (Cell Signaling, Beverly, MA). Signal was detected with enhanced chemiluminescence (GE Healthcare, Piscataway, NJ) following incubation with horseradish peroxidase-conjugated goat anti-rabbit secondary antibody (Santa Cruz Technology). Quantitation of Western films was performed using ImageJ software and results were normalized to GAPDH.

### Lactate and pyruvate assay

Assay of media lactate pyruvate levels were based on Noll [1], with modifications based on Galli [2]. Briefly, cell supernatants were heated to 65 °C for 1 hour and treated with 60 to 80 mesh Florisil (0.1 g/mL) to eliminate non-specific reduced nicotinamide adenine dinucleotide (NADH). Following centrifugation, supernatants were immediately assayed enzymatically for lactate and pyruvate by measuring NADH increase (339 nM) and reduction, respectively, in the presence of lactate dehydrogenase.

### Luciferase assays

Cell lysates were assayed for luciferase activity using Dual luciferase reporter assay system (Promega) and the Glomax multidetection system (Promega), according to manufacturer’s instructions. Firefly luciferase was normalized to Renilla luciferase.

### Ceramide Quantitation

Cells were washed once with PBS and resuspended in 100 µl of deionized water. Cells were lysed by 3 freeze/thaw cycles at 37 °C and dry ice ethanol bath. Protein content was measured. 10 µl of 0.1 nmol/µl C17:0 ceramide was added an internal standard. Ceramides were extracted by adding 750 µl of methanol and vortexing for 30 seconds followed by adding 2.5 ml of methyl-tert-butyl ether (MTBE). Following 1 hour of shaking, 625 µl of H_2_O was added to induce phase separation then centrifuged for 5 min at 900 g. The upper ceramide layer was taken and dried down under a nitrogen stream at room temperature. The dried product was reconstituted with 100 µl of ethanol/chloroform 2:1 (v/v).

### Flow injection ESI/MS/MS

Flow injection ESI/MS/MS analysis was performed using a Waters Xevo TQS electrospray tandem mass spectrometer fitted with an Acquity UPLC (Waters Corporation). The solvent was 5 mM ammonium formate in methanol/chloroform 2:1 (v/v). The ion source parameters were as follows: capillary voltage was set to 1.03 kV, source offset at 0 V, source temperature was 100 °C, desolvation temperature was 250 °C, and desolvation nitrogen flow was 600 L/hr. The multiple reaction monitoring (MRM) and precursor scan mode were used for data acquisition. The loss of one water fragment of the ceramide molecules became the dominant ion. Therefore an adjustment for different targeted ions for ceramides analysis was made in this study. The precursor ions and product ion transitions which were monitored for the ceramides were:

520.5-264 for C16:0-Cer, 534.5-264 for C17:0-Cer, 548.5-264 for C18:0-Cer, 576.5-264 for C20:0-Cer, 604.5-264 for C22:0-Cer, 630.5-264 for C24:1-Cer, and 632.5-264 for C24:0-Cer. NeoLynx 4.1 software (Waters Corporation) was used for calculations.

**Calculation**

$${{\rm{Mass}}}_{{\rm{unknown}}}\,:{{\rm{Mass}}}_{{\rm{internal}}{\rm{std}}}={{\rm{Conc}}}_{{\rm{unknown}}}\,:{\rm{Conc}}{{\rm{.}}}_{{\rm{internal}}{\rm{std}}}$$

$${\rm{Conc}}{{\rm{.}}}_{{\rm{unknown}}}=\frac{\frac{{{\rm{Mass}}}_{{\rm{unknown}}}}{{{\rm{Mass}}}_{{\rm{internal}}{\rm{std}}}}\times {\rm{Conc}}{{\rm{.}}}_{{\rm{internal}}{\rm{std}}}}{{\rm{Protein}}\,{\rm{Conc}}{\rm{.}}}$$

${\rm{Conc}}{{\rm{.}}}_{{\rm{internalstd}}}={\rm{0.1}}\,\mathrm{nmol}/\mu l$(10 µl internal standards was used).

[Protein Conc.] was in µg/µl.

$$\begin{array}{rcl}{{\rm{Conc}}}_{{\rm{unknown}}} & = & \frac{\frac{{{\rm{Mass}}}_{{\rm{unknown}}}}{{{\rm{Mass}}}_{{\rm{internal}}{\rm{std}}}}\times (0.1\,\mathrm{nmol}/\mu {\rm{l}})\times {\rm{10}}\,\mu {\rm{l}}}{[\mathrm{Protein}\,{\rm{Conc}}.]\,\mu g/\mu {\rm{l}}\times {\rm{100}}\,\mu {\rm{l}}}\\  & = & \frac{\frac{{{\rm{Mass}}}_{{\rm{unknown}}}}{{{\rm{Mass}}}_{{\rm{internal}}{\rm{std}}}}\times 1\,{\rm{nmol}}}{[\mathrm{Protein}\,{\rm{Conc}}.]\mu g\times {\rm{100}}}=\frac{\frac{{{\rm{Mass}}}_{{\rm{unknown}}}}{{{\rm{Mass}}}_{{\rm{internal}}{\rm{std}}}}\times 1\,{\rm{nmol}}}{\frac{[\mathrm{Protein}\,{\rm{Conc}}.]\mathrm{mg}\times {\rm{100}}}{{\rm{1000}}}}\\  & = & 10\times \frac{\frac{{{\rm{Mass}}}_{{\rm{unknown}}}}{{{\rm{Mass}}}_{{\rm{internal}}{\rm{std}}}}}{[\mathrm{Protein}\,{\rm{Conc}}.]\,}\mathrm{nmol}/\mathrm{mg}\,{\rm{protein}}\end{array}$$

### Statistical Analysis

Data are expressed as the mean ± standard error of the mean (SEM). T-test or analysis of variance (ANOVA) with post-hoc Newman-Keuls multiple comparison test (GraphPad Prism, La Jolla, CA) were used for statistical analysis. P < 0.05 is considered significant.

## Results

### Ethanol increases TGs and PLIN2 accompanied by β-oxidation inhibition in VL-17A cells

**Figure 1:**
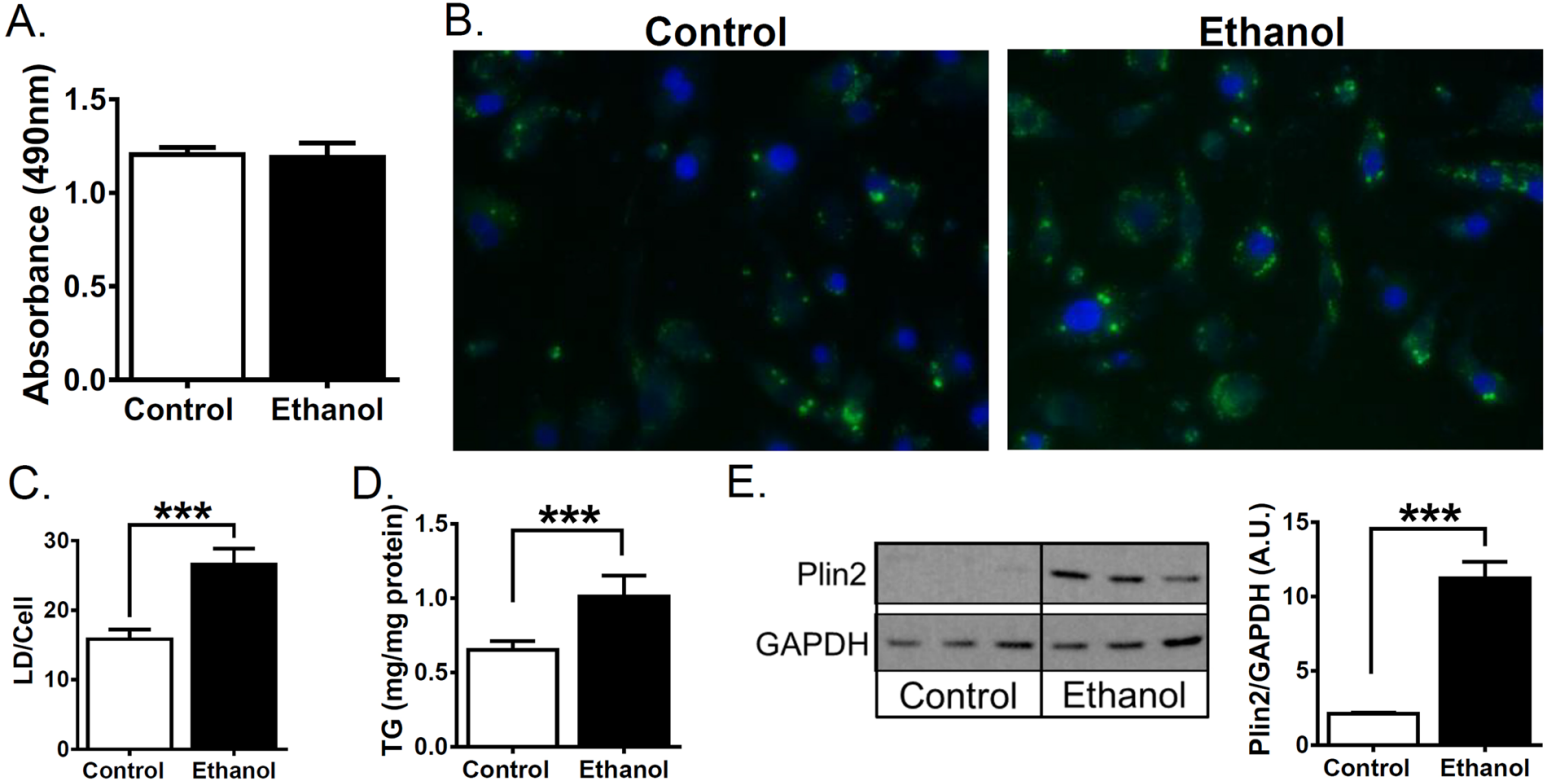
Ethanol treatment enhances lipid droplet accumulation, TG and PLIN2 expression in VL-17A human hepatoma cells. (**A–E**) VL-17A cells were treated for 48 h with control media or media supplemented with 100 mM ethanol. (**A**) Cell viability was assessed by CellTiter 96® Aqueous One Solution Cell Proliferation Assay. (**B**) Representative 10x images and quantitation from cells fixed and stained with lipophilic stain Nile Red (green) and DAPI (blue). (**C**) Quantitation of lipid droplets from stained cells. (**D**) TG determined in cell extracts (N = 5). (**E**) Cropped western blot images from different blots and quantitation of PLIN2 or GAPDH protein (N = 3) from cell lysates. Full length blots are in Supplementary Figure 1. Data presented as mean +/−SEM. ***p < 0.01 relative to control.

We and others have shown VL-17A cells accumulate LDs in response to ethanol and lipotoxic stimuli^26,39^. Based on our previous data, VL-17A cells were incubated in control or 100 mM ethanol media for 48 h. Under these conditions, ethanol treatment did not alter cell viability (Fig. 1A). Consistent with these cells being a good model for alcoholic steatosis, ethanol-treated cells accumulated significantly more LDs (Fig. 1B,C) and TG levels were elevated 1.6 fold (Fig. 1D). PLIN2, a lipid-droplet coating protein previously used as a marker of alcoholic steatosis^8^ was also increased in ethanol-treated cells (Fig. 1E).

Ethanol can promote TG accumulation by increasing FA uptake, by increasing DNL, reducing TG export in VLDL particles, or reducing FA oxidation. The relative contribution of these pathways to TG accumulation and PLIN2 upregulation in VL-17A cells is unknown.

**Figure 2:**
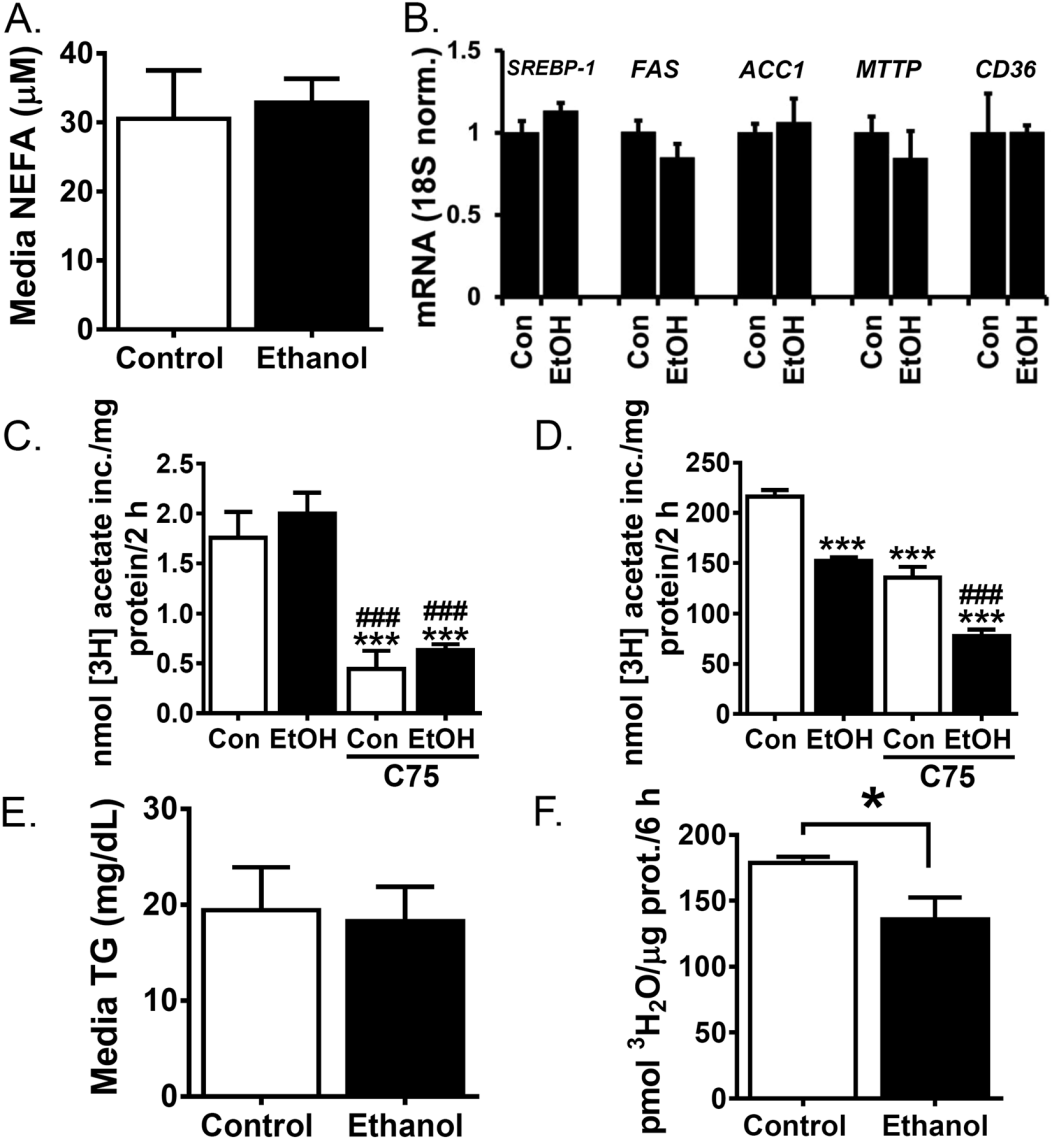
Ethanol treatment decreases fatty acid oxidation but not extracellular NEFA, TG or FA synthesis. (**A–F**) Cells were treated for 48 h with control media or media supplemented with 100 mM ethanol. (**A**) Cell supernatants were assessed for NEFA. (**B**) Isolated mRNA was assayed by real time RT-PCR for SREBP-1, FAS, ACC1, MTTP and CD36. VL-17A cells. (**C**) VL-17A cells were treated for 2 h with 500 µM 3H-acetate with or without the FAS inhibitor C75 (500 µM). Radiolabel incorporation into lipid soluble cell extracts was measured. (**D**) HepG2 cells were treated as in C. (**E**) Cell supernatants were assessed for TG. (**F**) Oleate oxidation was quantified by measuring ^3^H water liberation from ^3^H labelled oleate. Data presented as mean +/−SEM. *p < 0.05 relative to control, ***p < 0.01 relative to control, ^###^p < 0.01 relative to ethanol treated (N = 3–5).

We investigated media NEFA levels as a measure of FA uptake and found no difference in media NEFA levels between control- and ethanol-treated samples (Fig. 2A). Consistent with this finding, we found no effect of ethanol treatment on mRNA levels of the FA transporter, CD36 (Fig. 2B). We next measured mRNA levels of sterol regulatory element-binding protein 1 (SREBP-1), a transcription factor that drives production of a battery of genes that control DNL, such as FA synthase (FAS) and acetyl-coA carboxylase (ACC) 1. We found that ethanol treatment did not significantly alter mRNA levels of SREBP-1, FAS or ACC1 (Fig. 2B). To functionally assess DNL, we measured incorporation of ^3^H acetate into newly synthesized FAs. Although we noted inter-experiment variability^6^, in repeated studies we found ethanol treatment did not increase rates of acetate incorporation into FA (Fig. 2C). Treatment of control- and ethanol-treated cells with the FAS-specific inhibitor C75 blocked acetate incorporation (Fig. 2C), suggesting the observed incorporation is an accurate reflection of DNL activity. One explanation for the lack of an effect on DNL is that excess acetate due to ethanol oxidation dilutes the ^3^H-acetate label. To address this possibility, we repeated this experiment in HepG2 cells, which lack ethanol-metabolizing enzymes. We found ethanol-treated HepG2 cells also had decreased DNL (Fig. 2D), suggesting the lack of DNL induction observed in these cells is not due to label dilution. We next measured media TG as a surrogate marker of TG secretion. No TG was detected in media without cells (data not shown), suggesting measured TG was produced by VL-17A cells. We found that ethanol treatment had no effect on media TG levels (control 19.45 +/− 1.99 mg/dl, ethanol 18.30 +/− 1.59 mg/dl) (Fig. 2E). Consistent with these data, mRNA levels of microsomal triglyceride transfer protein (MTTP), a protein critical for VLDL assembly, were unaffected by ethanol (Fig. 2B).

In contrast to the negligible effect of ethanol on FA uptake, DNL or TG secretion, we found significant effects of ethanol on FA oxidation. Ethanol decreased oxidation of the long chain FA oleate by 25% (control 178.5 +/− 2.68, ethanol 135.7 +/−9.8 pmol H_2_O/µg dna/6 h, p = 0.013, Fig. 2F). These data suggest that ethanol-induced TG accumulation in VL-17A cells is due primarily to reductions in FA oxidation.

### Restoration of redox balance normalizes FA oxidation rates in ethanol-treated cells

**Figure 3:**
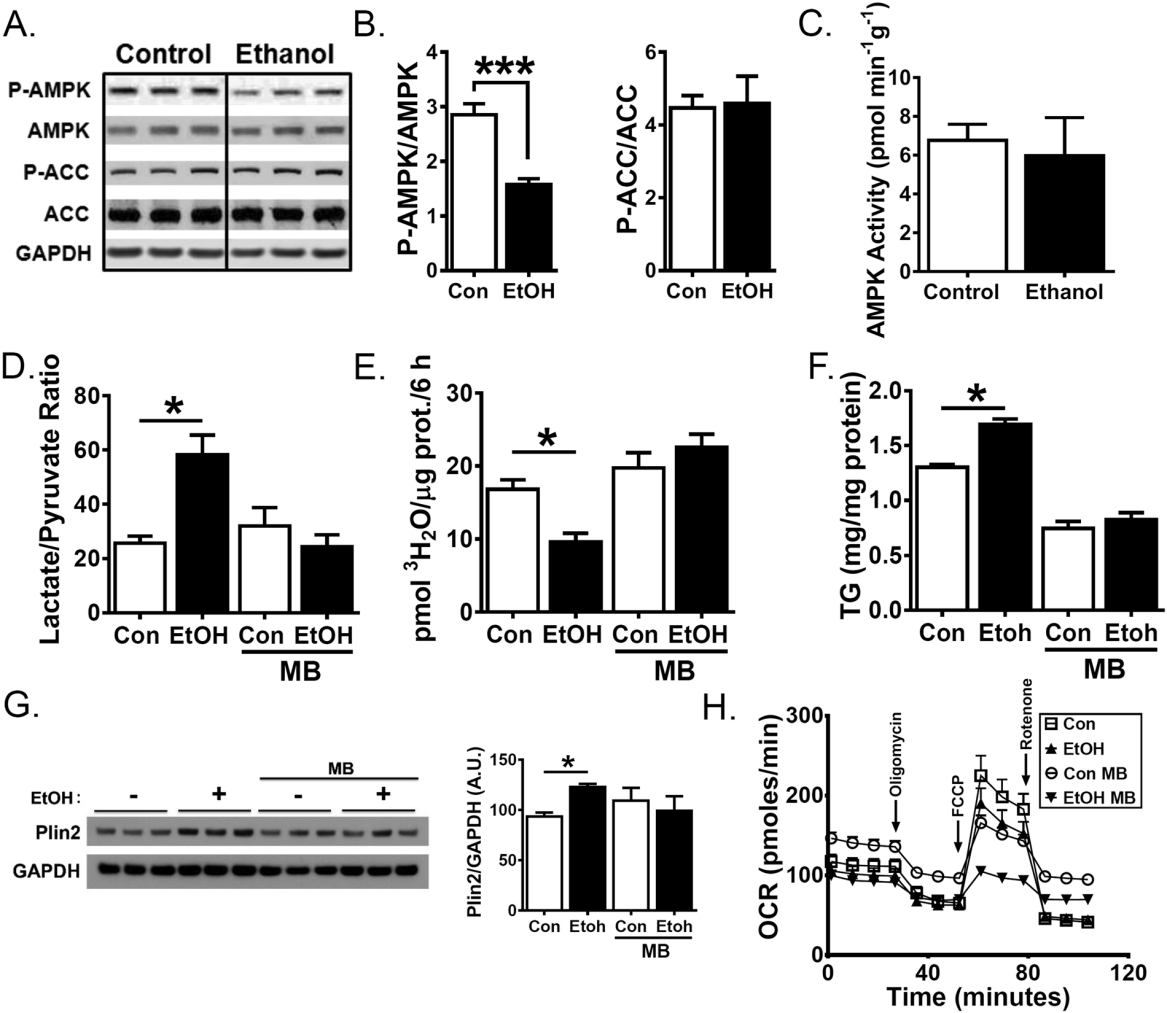
Restoring redox potential normalizes fatty acid oxidation and blocks TG accumulation in ethanol treated cells. (**A–F**) VL-17A cells were treated for 48 h with control media or media supplemented with 100 mM ethanol alone or with 10 µM methylene blue (MB) for 24 h (**D**–**G**). (**A**) Cropped western blot images for GAPDH, phosphorylated and total AMPK and ACC from different blots. Full length blots are in Supplementary Figure 1. (**B**) Densitometric quantitation of blots in A. (**C**) AMPK activity was measured in cell lysates by measuring ^32^P-phosphate transfer from radiolabeled ATP to the SAMS peptide. (**D**) Lactate/pyruvate ratio was determined enzymatically in cell supernatants. (**E**) Oleate oxidation was quantified by measuring ^3^H water liberation from ^3^H labelled oleate. (**F**) TG was measured in cell lysates. (**G**) Cropped western blot images from different blots and quantitation of PLIN2 and GAPDH protein levels (N = 3) from cell lysates. Full length blots are in Supplementary Figure 1. (**H**) Oxygen Consumption Rate was measured using Seahorse XF96 Analyzer during a mitochondrial stress test. 1 µM Oligomycin, 1 µM Carbonyl cyanide-4-(trifluoromethoxy)phenylhydrazone (FCCP), and a mix of 0.5 µM rotenone and antimycin A were added at the given times. Data presented as mean +/−SEM. *p < 0.05 relative to control, ***p < 0.01 relative to control, (N = 3–5).

We next sought to determine the mechanism by which ethanol treatment decreases FA oxidation in VL-17A cells. PPARα and AMPK are two molecular activators of FA oxidation known to be inhibited by ethanol^47,48^. PPARα is a transcription factor that stimulates production of genes important for FA oxidation, such as carnitine palmitoyltransferase 1 (CPT1) and malonyl-coenzyme A (CoA) decarboxylase (MCD). AMPK is activated by phosphorylation (pAMPK) and promotes FA oxidation by reducing the CPT1 inhibitor malonyl-CoA. It accomplishes this indirectly through inhibitory phosphorylation of the malonyl-CoA synthetic enzyme, ACC. Ethanol treatment did not alter mRNA levels of PPARα or its downstream targets, CPT1 and MCD, compared to control-treated VL-17A cells (Table 1). We next examined AMPK activity. Ethanol lowered AMPK phosphorylation by 45% compared with control (Fig. 3A,B). However, the observed pAMPK reduction did not correlate with AMPK functional measures including ACC phosphorylation (Fig. 3A,B) or kinase activity (Fig. 3C) in cell extracts. These data suggest FA oxidation reduction in ethanol-treated VL-17A cells is not due to inhibition of PPARα or AMPK.

**Table 1:** VL-17A cells were treated for 48 h with control- or ethanol-containing media supplemented with BSA, oleate, palmitate or C2 ceramide.

		BSA	Oleate		Palmitate		C2 Ceramide	
		AVG ± SEM	AVG ± SEM	*p*	AVG ± SEM	*p*	AVG ± SEM	*p*
Control	PPARα	1.00 ± 0.22	1.02 ± 0.10	1.00	0.82 ± 0.12	0.97	0.99 ± 0.18	0.98
	CPT1	1.00 ± 0.13	1.82 ± 0.07	0.57	0.86 ± 0.10	0.65	0.92 ± 0.09	0.77
	MCD1	0.99 ± 0.00	1.05 ± 0.11	0.99	0.69 ± 0.11	0.78	1.31 ± 0.39	0.95
	SREBP-1C	1.00 ± 0.09	1.53 ± 0.35	0.89	0.97 ± 0.13	0.99	1.18 ± 0.19	0.94
	FAS	1.00 ± 0.09	1.27 ± 0.10	0.23	1.31 ± 0.34	0.85	1.50 ± 0.14	0.10
	ACC1	1.00 ± 0.05	0.92 ± 0.12	0.96	0.69 ± 0.11	0.78	1.31 ± 0.19	0.33
Ethanol	PPARα	1.04 ± 0.07	1.02 ± 0.19	1.00	0.87 ± 0.08	0.97	2.86 ± 0.26	0.01
	CPT1	1.15 ± 0.12	2.14 ± 0.79	0.48	1.20 ± 0.09	0.78	2.03 ± 0.22	0.02
	MCD1	0.96 ± 0.08	1.01 ± 0.08	0.99	1.32 ± 0.43	0.78	1.28 ± 0.14	0.95
	SREBP-1C	1.24 ± 0.11	0.77 ± 0.12	0.89	1.00 ± 0.10	0.78	1.16 ± 0.20	0.94
	FAS	1.04 ± 0.21	1.09 ± 0.08	0.24	0.61 ± 0.20	0.80	0.99 ± 0.09	0.94
	ACC1	0.90 ± 0.14	1.02 ± 0.06	0.96	1.32 ± 0.43	0.78	1.36 ± 0.07	0.25

Isolated mRNA was reverse transcribed and transcript levels were determined using real-time RT-PCR and normalized to 18S RNA levels. Data presented as mean +/−SEM (N = 3–5). *p* values were calculated relative to BSA control by ANOVA.

We next investigated alcohol’s effects on NADH:NAD^+^ ratio, because ethanol oxidation is coupled to the reduction of NAD^+^ to NADH and this has been shown to inhibit FA oxidation^49^. The cellular NADH:NAD^+^ ratio is reflected in the lactate:pyruvate ratio^50^, so lactate and pyruvate were measured spectrophotometrically by enzymatic assays^51^. We verified that supernatant lactate:pyruvate ratios reflect cellular ratios (data not shown), consistent with previous reports^52^. Ethanol treatment increased the lactate:pyruvate ratio 2.3-fold (p < 0.05, Fig. 3D). To functionally address whether increased lactate:pyruvate ratio in ethanol-treated cells decreased FA oxidation, we treated cells with 10 µM methylene blue (MB), an electron acceptor capable of oxidizing NADH to NAD^+^. MB blocked the ethanol-induced increase in the lactate:pyruvate ratio (Fig. 3D) and restored FA oxidation levels in ethanol treated cells to control levels (Fig. 3E). FA oxidation restoration in ethanol-treated cells by MB also prevented increased TG and PLIN2 levels (Fig. 3F,G).

To test whether MB treatment works by enhancing mitochondrial function, we performed mitochondrial stress tests in the Seahorse XFe96 Analyzer. We found ethanol-treated cells have a non-statistically significant lowering in baseline mitochondrial respiration evidenced by reduced oxygen consumption rate (OCR) prior to inhibitor addition (Fig. 3H). Ethanol treatment also tended to decrease maximal respiratory rate observed after 1 µM FCCP addition. In the absence of MB, both control and ethanol-treated cells have similar contributions from non-mitochondrial respiration as indicated by OCR following the addition of 0.5 µM rotenone and antimycin A. In contrast, MB-incubated control cells have a higher basal oxygen consumption rate and lower maximal respiratory rate compared with both control and ethanol-treated cells. In the presence of ethanol, methylene blue cells have a significantly decreased basal oxygen consumption rate and maximal respiratory rate suggesting that the effects of methylene blue on fatty acid oxidation are not due to primary effects on mitochondrial respiration. Together, these data demonstrate that an increased NADH:NAD^+^ ratio mediates ethanol’s impairment of FA oxidation and subsequent TG accumulation in VL-17A cells.

### C2 ceramide restores FA oxidation in ethanol-treated VL-17A cells

**Figure 4:**
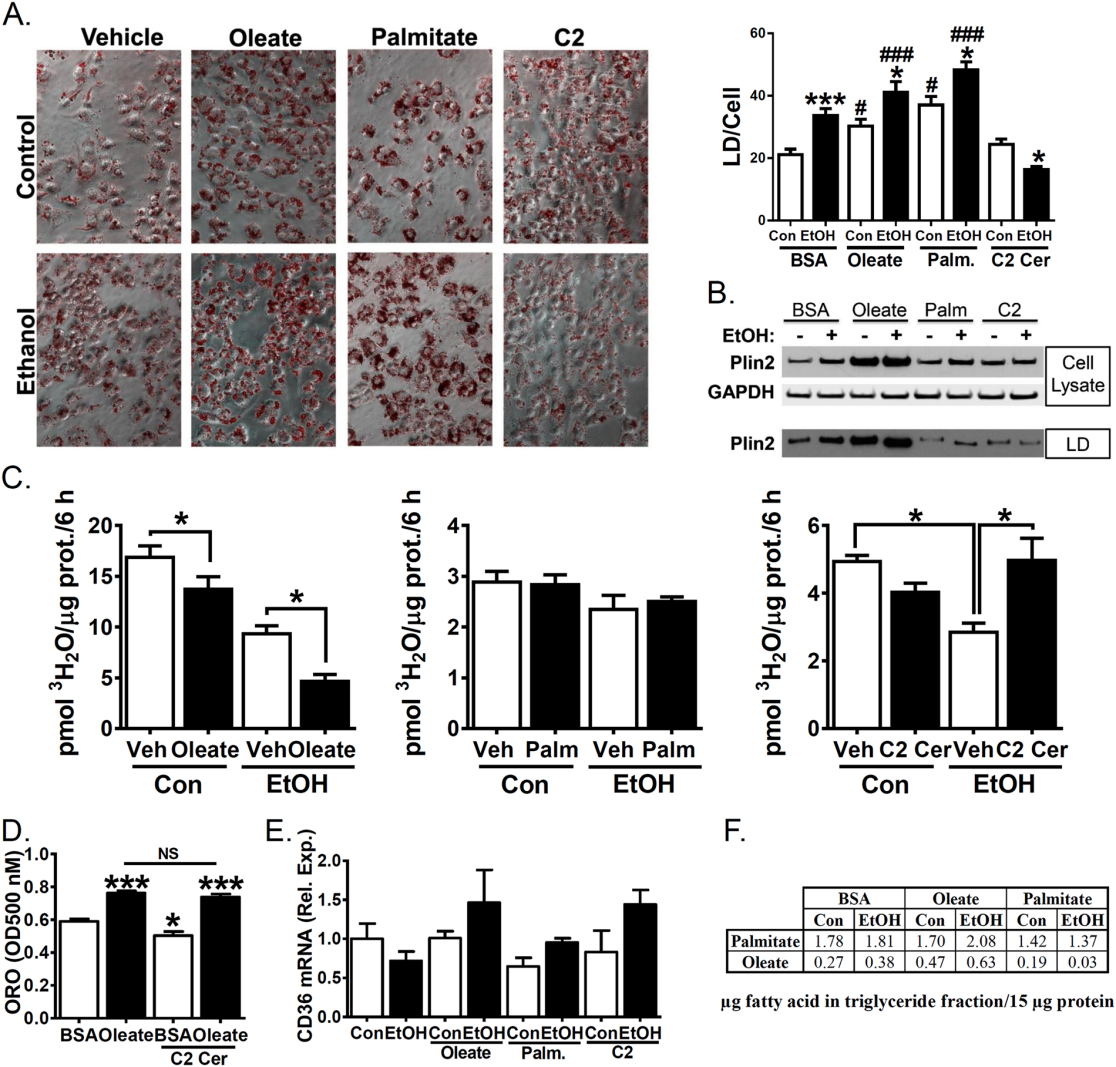
Co-treatment with C2 ceramide but not oleate or palmitate reverses ethanol-mediated reduction in fatty acid oxidation. (**A–F**) VL-17A cells were treated for 48 h with control- or 100 mM ethanol-containing media or supplemented with BSA (vehicle control), 100 µM oleate, 40 µM palmitate or 10 µM C2 ceramide. (**A**) Representative images and quantitation (N = 17–20) from cells fixed and stained with Oil Red O. (**B**) PLIN2 and GAPDH were measured by western blotting of whole cell lysates or isolated lipid droplets. Representative cropped western blot images from different blots shown. Full-length blots are in Supplementary Figure 1. (**C**) Oleate oxidation was quantified by measuring ^3^H water liberation from ^3^H labelled oleate (N = 5). (**D**) Oil red O staining in fixed cells was quantified by elution in isopropanol followed by optical density reading at 500 nM. (**E**) Isolated mRNA was assayed by real time RT-PCR for CD36. (**F**) LC-MS/MS analysis of FA composition of triglyceride. Listed values are µg fatty acid in triglyceride fraction/15 µg protein. Data presented as mean +/−SEM. *p < 0.05 relative to BSA Con, ***p < 0.01 relative to BSA Con, ^###^p < 0.01, ^#^p < 0.05 compared to BSA Con with same exogenous lipid.

Given the additive effect of ethanol and lipids on development of hepatic steatosis^13,14^ we systematically examined the effects of exogenous lipids on ethanol-induced TG accumulation in VL-17A cells. We selected two common dietary FAs, oleate and palmitate, as representative unsaturated and saturated FA, respectively. We also tested the effects of the sphingolipid ceramide, as our previous studies have implicated ceramides in the pathophysiology of alcoholic liver disease^7,15^. We used 100 µM oleate and 40 µM palmitate based on normal physiological levels in human serum^53^. We used 10 µM of the short-chain ceramide, C2 ceramide, as it causes minimal toxicity as assessed by cell morphology (data not shown) and is readily cell permeable^27^. All lipids were conjugated to BSA prior to addition to the media, and BSA alone was used as a vehicle control. Cells incubated with either oleate or palmitate had increased Oil Red O staining compared with cells incubated in control media or ethanol alone (Fig. 4A). Fatty acid treatment also increased PLIN2 expression in whole cell lysates and isolated lipid droplets (Fig. 4B). C2 ceramide did not increase Oil Red O staining (Fig. 4A). Unlike exogenous FAs, co-incubation with ethanol and C2 ceramide decreased Oil Red O staining relative to C2 ceramide alone (Fig. 4A) while PLIN2 levels were similar (Fig. 4B).

Co-incubation of ethanol and exogenous lipids had no effect on DNL gene expression (Table 1) or extracellular TG levels (data not shown). Next, we examined the combined effects of ethanol and exogenous lipids on FA oxidation rates. Co-incubation with oleate further diminished FA oxidation in ethanol cells while co-incubation of ethanol and palmitate had no effect on FA oxidation (Fig. 4C). C2 ceramide did not alter rates of FA oxidation in control cells (Fig. 4C), but when co-incubated with ethanol increased FA oxidation rates by 75% (p = 0.011) relative to ethanol alone (Fig. 4C). To determine if the protective effects of C2 on ethanol-mediated lipid accumulation were specific to C2 ceramide, we examined whether co-incubation with C2 ceramide could also inhibit oleate-induced TG accumulation. To that end, we performed quantitation of eluted Oil Red O cell staining and found that cells similarly accumulated Oil Red O when treated with oleate alone or in combination with C2 ceramide (Fig. 4D). We next examined whether FA treatment increased uptake through transcriptional regulation of the uptake receptor CD36 and found CD36 mRNA levels were unaffected by exogenous lipid treatment or ethanol treatment (Fig. 4E).

In an effort to further understand the fate of exogenous lipids and ceramides, we performed liquid chromatography and tandem mass spectrometry to determine the FA composition of LD TG content in response to incubation with dietary fatty acids; and also performed mass spectrometric analysis of ceramide species of differing chain lengths and saturation in both whole cell lysates and LDs. We found oleate treatment increased oleate incorporation into LD TG while palmitate did not increase palmitate levels in LD TG (Fig. 4F). In whole cell lysates, ethanol-treated cells had decreased ceramide species Cer 16:0, 22:0, 24:0 and 24:1 (Supp. Table 1). Decreases in Cer 16:0, 22:0, 24:0 were also observed in LDs isolated from EtOH-treated cells (Supp. Table 2). Oleate treatment increased Cer 6:0, 8:0 and lowered Cer 24:0 (Supp. Table 1) relative to control-treated cells, but these changes were not evidenced in the LD fraction (Supp. Table 2). Palmitate treatment increased Cer 6:0, 8:0, 16:0, 18:0 and 22:3 (Supp. Table 1), with the elevated Cer 6:0, 8:0, 16:0 and 22:3 evidenced in the LD fraction (Supp. Table 2).

### C2 ceramide and ethanol co-incubation increases FA oxidation indirectly through PPARα

We next sought to investigate the mechanism by which co-incubation of exogenous lipids with ethanol alters FA oxidation. Similar to the aforementioned studies, we examined gene expression of oxidative genes using RT-PCR analysis of PPARα, MCD, CPT1, and ACC. Our results show that oleate and palmitate, either alone or in combination with ethanol have no effect on PPARα, MCD1, CPT1, or ACC mRNA levels (Table 1). In contrast, although C2 ceramide alone had no effect on these mRNA levels and ethanol alone lowered oxidation by a PPARα-independent mechanism, C2 ceramide in combination with ethanol caused a three-fold increase in PPARα mRNA and two-fold increase in CPT1 mRNA relative to control treated cells (Table 1).

**Figure 5:**
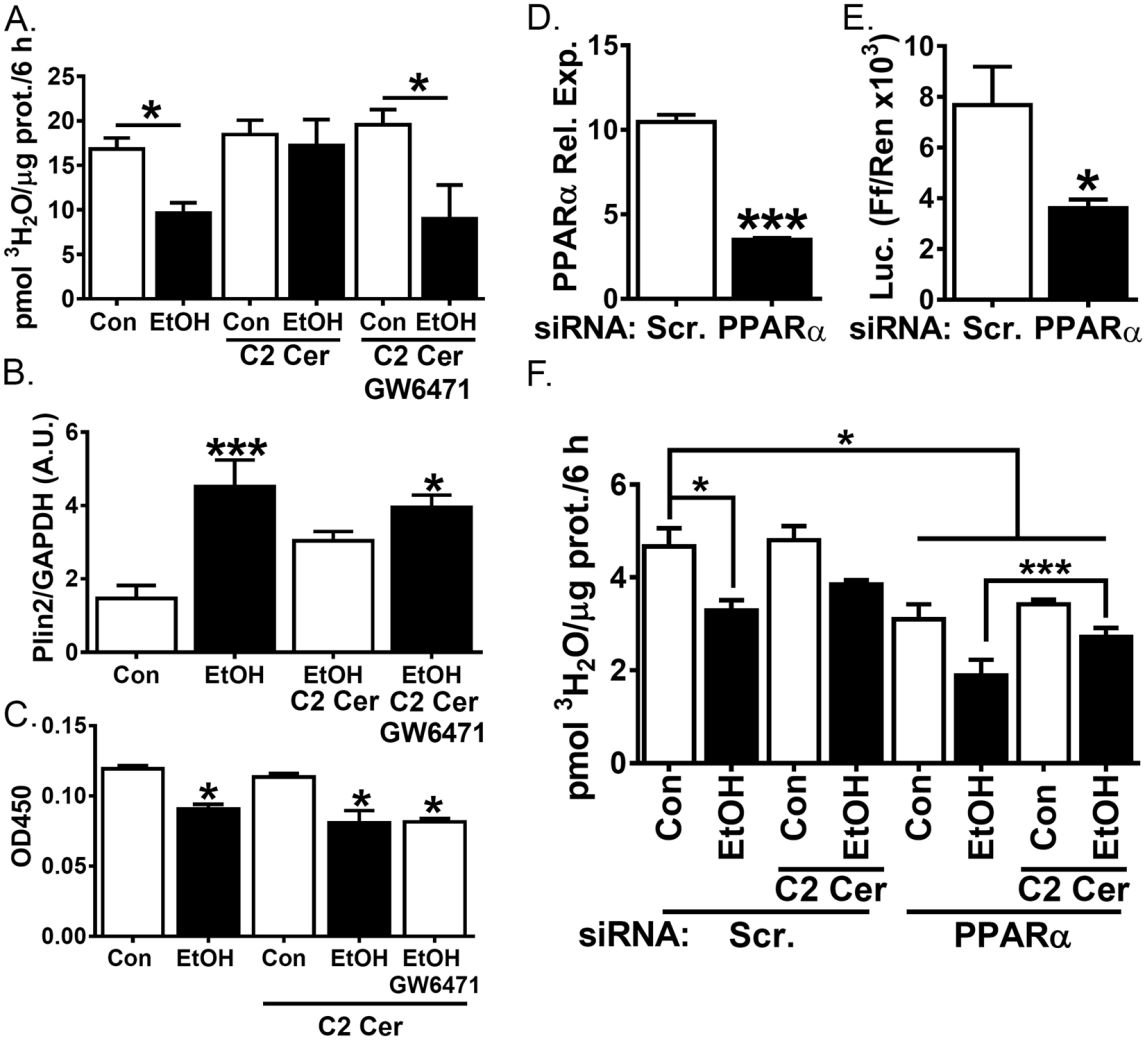
PPARα antagonist GW6471 restores ethanol-mediated inhibition of FA oxidation and increased PLIN2 expression in C2 ceramide treated cells. (**A–F**) VL-17A cells were treated for 48 h with control or ethanol (100 mM)-containing media supplemented with 10 µM C2 ceramide. (**A–C**) cells were treated with 5 µM GW6471 for 24 h. (**A**) Oleate oxidation was quantified by measuring ^3^H water liberation from ^3^H labelled oleate (N = 5). (**B**) Densitometric quantification of PLIN2 and GAPDH measured in cell lysates by western blot (N = 3). (**C**) PPARα binding in nuclear extracts were quantified by transcription factor binding assay. (**D–F**) cells were transfected with scramble or PPARα siRNA 5 days before ethanol treatment. (**D**) PPARα mRNA was assayed by real time RT-PCR. (**E**) Firefly and renilla luciferase activity was assayed in cell extracts 48 h following transfection with the Cignal PPAR Reporter plasmid. (**F**) Oleate oxidation was quantified as in A. Data presented as mean +/−SEM. *p < 0.05 relative to control, ***p < 0.01 relative to control.

To address whether the increase in FA oxidation in VL-17A cells co-incubated with ethanol and C2 ceramide is due to PPARα activation, we utilized a pharmacological PPARα antagonist, GW6471. Pharmacologic PPARα inhibition prevented the C2 ceramide-stimulated FA oxidation in ethanol-incubated VL-17A cells (Fig. 5A) and increased PLIN2 protein levels (Fig. 5B). We next measured PPARα activation by assaying its DNA-binding activity in nuclear extracts. We found that ethanol treatment decreased PPARα activity, however, this was not restored upon co-stimulation with ethanol and C2 ceramide (Fig. 5C). Next, we measured FA oxidation in cells treated with PPARα siRNA. We found siRNA treatment lowered PPARα RNA (Fig. 5D) and activity (Fig. 5E). However, co-treatment with C2 ceramide and ethanol similarly restored FA oxidation relative to cells treated with ethanol alone (Fig. 5F), suggesting PPARα is dispensable for this effect. Therefore, we conclude that there is an indirect effect of the observed effects of C2-ceramide and ethanol co-incubation on PPARα gene upregulation.

## Discussion

Alcoholic steatosis is the earliest histopathologic phenotype of patients with ALD, is present in the majority of patients who overconsume alcohol, and is a risk factor for developing advanced stages of ALD such as steatohepatitis and fibrosis^54^. Approximately 81% of patients relapse after abstaining from alcohol^54^, and there are no pharmacotherapies that target early stage disease. We have previously reported in a PLIN2 knock-out mouse model that reducing hepatocellular LD storage capacity not only prevents alcoholic steatosis but also improves glucose tolerance and insulin resistance (clinical factors associated with the development of advanced ALD)^7^, suggesting that modulation of steatosis itself may lower the risk of advanced ALD. Here, we sought to establish the relative contribution of pathways that promote LD accumulation using a human hepatocellular *in vitro* model of alcoholic steatosis. Because obesity and overnutrition contribute to the severity and progression of ALD^9^, we also investigated mechanisms of how exogenous lipids modify ethanol’s effects on hepatocellular LD homeostasis. We provide evidence that ethanol primarily promotes hepatocellular accumulation in VL-17A cells by increasing the NADH:NAD^+^ ratio which impairs FA β-oxidation. We demonstrate further that this inhibitory effect of ethanol on oxidation is exacerbated by the unsaturated FA oleic acid and ameliorated by C2 ceramide meditated indirectly by PPARα gene upregulation.

The major pathways that contribute to hepatocellular LD triglyceride accumulation are increased FA uptake, increased *de novo* lipogenesis, diminished triglyceride export as VLDL or decreased FA oxidation. We systematically examined each of these potential contributors in human hepatoma VL-17A cells. We established that in VL-17A cells incubated with ethanol there is an increase in cellular LDs and triglyceride content and an upregulation of PLIN2 protein, thus replicating both human^55^ and experimental alcoholic steatosis^8^. Our study demonstrates that ethanol increases LD accumulation in these human hepatoma cells primarily by inhibiting FA oxidation as measured by radiolabeled water release from tritiated oleic acid. This finding is consistent with several prior reports that have demonstrated ethanol’s impairment of FA oxidation is a major factor in the development of hepatic steatosis^5,56–58^. It is likely that the increased FAs resulting from lowered FA oxidation are esterified into triglycerides^57^ and stored in LDs.

The oxidation of ethanol to acetaldehyde and acetate is coupled to the reduction of NAD^+^ to NADH, and NAD^+^ is required to drive mitochondrial FA oxidation. In VL-17A cells, we demonstrate that ethanol’s increase of the NADH:NAD^+^ ratio is required for the impairment of FA oxidation while normalization of this ratio with the anti-oxidant MB restores normal FA oxidation rates and hepatocellular triglyceride levels, further demonstrating the critical role of impaired FA oxidation in ethanol-induced hepatocellular triglyceride accumulation. When we examined mitochondrial function, we observed methylene blue incubated control cells have a higher basal oxygen consumption rate and lower maximal respiratory rate compared with both control and ethanol-treated cells. In the presence of ethanol, methylene blue cells have a significantly diminished basal oxygen consumption rate and maximal respiratory rate suggesting that the effects of methylene blue on fatty acid oxidation are not due to primary effects on mitochondrial respiration. Rather, our results support a contribution from non-mitochondrial respiration in methylene blue treated control and ethanol cells. We, therefore, conclude that normalization of the redox potential lowers cellular TG independent of an increase in mitochondrial respiration. We hypothesize that such normalization may indirectly increase the availability of substrate for mitochondrial transfer, fatty acid oxidation and/or upregulate enzymes involved in non-mitochondrial respiration such as NADPH oxidases. Our findings in human hepatoma cells build upon those of Galli *et al*. who show that changes in redox potential are sufficient to drive triglyceride accumulation in ADH-transfected cervical cancer HeLa cells^52^. We recognize, however, that systemic MB given to alcohol-fed rats partially normalizes the NADH:NAD^+^ ratio but does not prevent hepatic triglyceride accumulation^59^. Therefore, our data *vis*-à-*vis* this study by Ryle, *et al*. support the hypothesis that both direct effects of ethanol on hepatic FA oxidation as well as systemic effects of ethanol contribute to hepatic triglyceride accumulation *in vivo*.

To our surprise, we did not observe an upregulation of the key *de novo* lipogenic nuclear receptor SREBP1 or its downstream targets, ACC and FAS, nor did we observe an increase in acetate incorporation (a physiologic measure of FA synthesis) in ethanol-incubated cells compared with controls. These results differ from those reported by You, *et al*. who used a luciferase reporter gene construct to demonstrate in rat hepatoma cell lines that ethanol increases SREBP1 reporter gene activity. They also found an upregulation of SREBP1 and downstream lipogenic enzymes in mice fed a low fat, high carbohydrate, 28.5% total caloric content ethanol diet^60^. The discrepancy between these studies reflects one of the longstanding challenges of understanding the metabolic effects of ethanol in experimental models. Given the complex interaction between alcohol, nutrients, the liver and extrahepatic tissues, studies have confirmed^61–63^, refuted^64,65^, or been unable to draw conclusions^5,6,66,67^ about the relative contribution of FA synthesis to alcoholic steatosis. Because we also suspect that there is an interaction between ethanol and nutrients on hepatic lipid metabolism, we analyzed the combined effect of ethanol and exogenous lipids on hepatocellular lipid metabolism.

Indeed, our data demonstrate a complex interaction between ethanol and exogenous lipids in both the promotion and amelioration of FA oxidation and LD accumulation. VL-17A cells co-incubated with ethanol and the unsaturated FA oleate exacerbates LD accumulation, increases triglyceride content, and further impairs FA oxidation relative to ethanol-incubated cells. Co-incubation with ethanol and the saturated FA palmitate has no effect on ethanol’s inhibition of FA oxidation, while co-incubation with C2 ceramide significantly decreases LD accumulation and restores FA oxidation to control levels. The finding that palmitate increases LDs without altering FA oxidation suggests palmitate increases TG through another mechanism. Possibilities include effects of ethanol on palmitate elongation and/or esterification. Indeed, although our own studies have not examined these specific mechanisms in the context of palmitate, ethanol has been demonstrated previously to have nearly equivalent effects on palmitate esterification and oxidation (without significant contribution from palmitate elongation)^57^.

Our findings of the effects of exogenous FA support *in vitro* studies suggesting a role for dietary or adipose-derived free FAs in the pathogenesis of alcoholic steatosis^3^. Our data also support *in vivo* data establishing a role of dietary FA in experimental ALD. For example, Nanji, *et al*. reported that administration of a saturated fat, palmitate-rich diet to alcohol-fed rats nearly resolved hepatic steatosis and lipid peroxidation, while a polyunsaturated FA diet failed to improve alcohol liver injury^68^. To our knowledge, however, our study is the first report of a protective effect of C2 ceramide on ethanol-mediated inhibition of hepatic FA oxidation and lipid droplet accumulation.

To date, few studies exist regarding the role of short chain ceramides on hepatocellular lipid biology. C2 ceramide is a short chain ceramide that downregulates methionine adenosyltransferase, a key regulator of hepatic regeneration, in primary rat hepatocytes and rat hepatoma cultured cells^69^. Most studies examining the role of ceramides in hepatocellular biology (including our own in an experimental mouse model of alcoholic steatosis^7,23^) have suggested a pathogenic role of long-chain ceramides^70^. Although C2 ceramides can be hydrolyzed and re-acylated to form these longer chain species in adenocarcinoma cells and keratinocytes^30,71^, the role of short-chain ceramides *per se* in liver biology is not well understood and has not been established previously in the context of ethanol-mediated hepatocellular lipid metabolism. Consistent with our recently published work demonstrating positive regulation of *PLIN2* gene stability by the ceramide synthetic enzyme ceramide synthase 6^26^, C2 ceramide upregulates PLIN2. However, C2 ceramide alone has no effect on FA oxidation or PPARα in VL-17A cells yet is able to reverse ethanol’s impairment of FA oxidation via an indirect PPARα-mediated mechanism. These results indicate that there is a specific interaction between C2 ceramide and ethanol in hepatocellular lipid biology.

The present study adds to a growing number of studies demonstrating both positive and negative feedback between PPAR family members and the cellular ceramide pool. The PPARα agonist fenofibrate increases ceramide content and the oxidative enzyme Cpt1^72^. Studies in H9c2 cells show PPARδ activation with docosahexaenoic acid increases ceramide levels and cellular toxicity^73^. Pharmacologic ceramide reduction prevents ceramide accumulation and toxicity in this model. Others have shown that PPAR activation can also lower ceramide levels. Treatment of ethanol-fed rats with a PPARδ agonist decreases hepatic ceramide levels and enhances insulin sensitivity^74^. There is also evidence that ceramides function directly as PPAR agonists. In a study examining expression of ABCA12, a glucosylceramide transporter important for keratinocyte barrier function, Jiang and colleagues demonstrate that C2 and C6 ceramides dose-dependently stimulate transcription of PPARδ, but not PPARα or PPARγ^75^. This in turn increases ABCA12 mRNA levels, which can be enhanced by blocking enzymes that metabolize ceramide, and inhibited by siRNA knockdown of PPARδ. Together, these data suggest that the relationship between PPAR activation and cellular ceramides is complex and context-specific, as PPARs can both increase and decrease ceramide levels, and PPARs can be directly activated by ceramides.

In summary, we aimed to investigate how ethanol and lipid co-factors impact hepatocellular lipid biology using our *in vitro* model of alcoholic steatosis in VL-17A cells. We show that impairment of FA oxidation is the major determinant of LD accumulation in ethanol-incubated VL-17A cells and that ethanol has differential interactions with exogenous lipids in hepatocellular lipid homeostasis. The interaction of ethanol with the unsaturated FA oleate worsens steatosis and FA oxidation while the short chain ceramide C2 remedies the ethanol-mediated FA oxidative defects at least in part via a PPARα-mediated mechanism. These findings provide evidence that the earliest stage of ALD, alcoholic steatosis, can be modulated experimentally by lipids. These observations may inform the design of novel strategies to help patients at risk for progression to more advanced stages of ALD.

## Electronic supplementary material

Dataset 1
