# Supplementary material for: Ethanol and C2 ceramide activate fatty acid oxidation in human hepatoma cells
Source: Sci Rep. 2018 Aug 27;8:12923. doi: 10.1038/s41598-018-31025-0 (PMC6110824; doi:10.1038/s41598-018-31025-0)

Ethanol and C2 ceramide activate fatty acid oxidation in human hepatoma cells

Jason M. Correnti, Lauren Gottshall, Annie Lin, Bianca Williams, Amanke Oranu, James Beck, Jie Chen

Michael J. Bennett and Rotonya M. Carr.

Supplementary Figures

Supplementary Table 1: *Ceramide Quantitation in whole cell extracts by mass spectrometry.* VL-17A cells were treated for 48 h with control- or 100 mM ethanol-containing media or supplemented with BSA (vehicle control),100 µM oleate, 40 µM palmitate or 10 µM C2 ceramide. Protein normalized samples were loaded onto LC-MS/MS. Listed concentrations are in nM. Listed p values compare control and ethanol samples within same lipid treatment. *: p<0.05, N=3

	**BSA**			**Oleate**			**Palmitate**			**C2 Ceramide**		
	**Control**	**Ethanol**		**Control**	**Ethanol**		**Control**	**Ethanol**		**Control**	**Ethanol**	
	AVG ± SEM	AVG ± SEM	*p*	AVG ± SEM	AVG ± SEM	*p*	AVG ± SEM	AVG ± SEM	*p*	AVG ± SEM	AVG ± SEM	*p*
**Cer C2:0**	13.3 ± 8.8	16.7 ± 3.3	0.74	23.3 ± 3.3	26.7 ± 12.0	0.80	20.0 ± 5.8	6.7 ± 6.7	0.21	60.0 ± 5.8*	73.3 ± 37.6	0.74
**Cer C4:0**	13.3 ± 8.8	10.0 ± 10.0	0.81	13.3 ± 3.3	13.3 ± 3.3	1.00	13.3 ± 8.8	6.7 ± 3.3	0.52	26.7 ± 6.7	13.3 ± 3.3	0.15
**Cer C6:0**	23.3 ± 3.3	16.7 ± 8.8	0.52	53.3 ± 8.8*	26.7 ± 13.3	0.17	36.7 ± 3.3*	40.0 ± 20.8	0.88	26.7 ± 3.3	20.0 ± 15.3	0.69
**Cer C8:0**	23.3 ± 3.3	16.7 ± 8.8	0.52	53.3 ± 8.8*	26.7 ± 13.3	0.17	36.7 ± 3.3*	40.0 ± 20.8	0.88	26.7 ± 3.3	20.0 ± 15.3	0.69
**Cer C10:0**	20.0 ± 5.8	26.7 ± 6.7	0.49	13.3 ± 3.3	20.0 ± 0.0	0.12	13.3 ± 3.3	16.7 ± 3.3	0.52	33.3 ± 8.8	20.0 ± 5.8	0.27
**Cer C12:0**	3.3 ± 3.3	6.7 ± 3.3	0.52	13.3 ± 3.3	10.0 ± 0.0	0.37	30.0 ± 11.5	23.3 ± 8.8	0.67	16.7 ± 8.8	16.7 ± 6.7	1.00
**Cer C14:0**	0.0 ± 0.0	16.7 ± 8.8	0.13	6.7 ± 3.3	10.0 ± 0.0	0.37	23.3 ± 18.6	10.0 ± 5.8	0.53	10.0 ± 5.8	16.7 ± 6.7	0.49
**Cer C14:1**	20.0 ± 5.8	16.7 ± 6.7	0.72	16.7 ± 6.7	10.0 ± 0.0	0.37	3.3 ± 3.3	6.7 ± 3.3	0.52	10.0 ± 5.8	16.7 ± 3.3	0.37
**Cer C16:0**	1363.3 ± 150.7	853.3 ± 67.7	0.04	1163.3 ± 66.9	730.0 ± 32.1*	0.00	1786.7 ± 117.2*	823.3 ± 39.3*	0.00	1426.7 ± 112.0	730.0 ± 30.6*	0.00
**Cer C16:1**	120.0 ± 25.2	70.0 ± 10.0	0.14	60.0 ± 32.1	80.0 ± 11.5	0.59	83.3 ± 27.3	83.3 ± 18.6	1.00	100.0 ± 11.5	100.0 ± 10.0	1.00
**Cer C18:0**	113.3 ± 13.3	93.3 ± 14.5	0.37	153.3 ± 50.4	110.0 ± 0.0	0.44	166.7 ± 13.3*	80.0 ± 20.0	0.02	196.7 ± 16.7*	110.0 ± 10.0	0.01
**Cer C18:1**	23.3 ± 8.8	16.7 ± 8.8	0.62	40.0 ± 5.8	20.0 ± 11.5	0.20	53.3 ± 12.0	16.7 ± 3.3	0.04	30.0 ± 10.0	26.7 ± 6.7	0.80
**Cer C18:2**	6.7 ± 3.3	16.7 ± 3.3	0.10	13.3 ± 3.3	3.3 ± 3.3	0.10	10.0 ± 10.0	13.3 ± 3.3	0.77	20.0 ± 5.8	10.0 ± 10.0	0.44
**Cer C20:0**	1880.0 ± 251.5	1506.7 ± 59.3	0.22	1583.3 ± 21.9	1420.0 ± 23.1	0.01	2130.0 ± 167.7	1630.0 ± 147.3*	0.09	1436.7 ± 718.4	2050.0 ± 240.1	0.46
**Cer C20:1**	46.7 ± 46.7	53.3 ± 27.3	0.91	23.3 ± 23.3	70.0 ± 5.8	0.12	76.7 ± 39.3	43.3 ± 21.9	0.50	100.0 ± 5.8	16.7 ± 16.7	0.01
**Cer C20:2**	20.0 ± 15.3	20.0 ± 5.8	1.00	16.7 ± 8.8	6.7 ± 3.3	0.35	33.3 ± 13.3	16.7 ± 3.3	0.29	3.3 ± 3.3	20.0 ± 10.0	0.19
**Cer C22:0**	356.7 ± 23.3	233.3 ± 26.0	0.02	253.3 ± 31.8	253.3 ± 29.6	1.00	420.0 ± 15.3	206.7 ± 24.0	0.00	263.3 ± 24.0*	206.7 ± 21.9*	0.16
**Cer C22:1**	80.0 ± 34.6	76.7 ± 3.3	0.93	86.7 ± 12.0	70.0 ± 5.8	0.28	103.3 ± 8.8	63.3 ± 14.5	0.08	116.7 ± 27.3	96.7 ± 31.8	0.66
**Cer C22:2**	36.7 ± 6.7	33.3 ± 8.8	0.78	30.0 ± 5.8	23.3 ± 6.7	0.49	53.3 ± 8.8	43.3 ± 3.3	0.35	20.0 ± 5.8	20.0 ± 10.0	1.00
**Cer C22:3**	13.3 ± 3.3	13.3 ± 3.3	1.00	16.7 ± 6.7	3.3 ± 3.3	0.15	36.7 ± 6.7*	20.0 ± 5.8	0.13	13.3 ± 3.3	16.7 ± 12.0	0.80
**Cer C22:4**	6.7 ± 3.3	10.0 ± 5.8	0.64	20.0 ± 10.0	6.7 ± 3.3	0.27	10.0 ± 5.8	6.7 ± 3.3	0.64	3.3 ± 3.3	3.3 ± 3.3	1.00
**Cer C24:0**	583.3 ± 27.3	420.0 ± 15.3	0.01	436.7 ± 28.5*	330.0 ± 43.6*	0.11	496.7 ± 31.8	323.3 ± 18.6*	0.01	523.3 ± 29.6	320.0 ± 25.2*	0.01
**Cer C24:1**	1070.0 ± 25.2	656.7 ± 57.0	0.00	1163.3 ± 31.8	643.3 ± 60.6*	0.00	1296.7 ± 121.3	616.7 ± 54.6*	0.01	1006.7 ± 127.2	660.0 ± 80.0*	0.08
**Cer C24:2**	73.3 ± 14.5	53.3 ± 12.0	0.35	73.3 ± 16.7	50.0 ± 5.8	0.26	103.3 ± 14.5	53.3 ± 14.5	0.07	50.0 ± 10.0	46.7 ± 17.6	0.88
**Cer C24:3**	16.7 ± 6.7	3.3 ± 3.3	0.15	26.7 ± 13.3	3.3 ± 3.3	0.16	26.7 ± 6.7	13.3 ± 6.7	0.23	16.7 ± 12.0	20.0 ± 15.3	0.87
**Cer C24:4**	6.7 ± 6.7	3.3 ± 3.3	0.68	10.0 ± 5.8	6.7 ± 6.7	0.72	10.0 ± 0.0	0.0 ± 0.0	0.00	3.3 ± 3.3	0.0 ± 0.0	0.37
**Cer C26:0**	26.7 ± 14.5	26.7 ± 12.0	1.00	40.0 ± 15.3	16.7 ± 3.3	0.21	36.7 ± 12.0	20.0 ± 11.5	0.37	23.3 ± 8.8	50.0 ± 15.3	0.21
**Cer C26:1**	33.3 ± 13.3	20.0 ± 5.8	0.41	26.7 ± 3.3	20.0 ± 10.0	0.56	40.0 ± 5.8	13.3 ± 13.3	0.14	30.0 ± 5.8	26.7 ± 6.7	0.72
**Cer C26:2**	6.7 ± 3.3	20.0 ± 5.8	0.12	16.7 ± 3.3	13.3 ± 8.8	0.74	16.7 ± 8.8	6.7 ± 3.3	0.35	16.7 ± 3.3	10.0 ± 5.8	0.37
**Cer C26:3**	6.7 ± 3.3	3.3 ± 3.3	0.52	13.3 ± 3.3	6.7 ± 3.3	0.23	20.0 ± 10.0	13.3 ± 6.7	0.61	6.7 ± 6.7	20.0 ± 11.5	0.37
**Cer C26:4**	60.0 ± 11.5	33.3 ± 12.0	0.18	23.3 ± 12.0	43.3 ± 12.0	0.30	60.0 ± 17.3	26.7 ± 3.3*	0.13	40.0 ± 10.0	56.7 ± 3.3	0.19

Supplementary Table 2: *Ceramide Quantitation in isolated lipid droplets by mass spectrometry.* VL-17A cells were treated for 48 h with control- or 100 mM ethanol-containing media or supplemented with BSA (vehicle control),100 μM oleate, 40 μM palmitate or 10 μM C2 ceramide. Lipid droplets were isolated, protein normalized and loaded onto LC-MS/MS. Listed concentrations are in nM.

	BSA		Oleate		Palmitate		C2 Ceramide	
	**Control**	**Ethanol**	**Control**	**Ethanol**	**Control**	**Ethanol**	**Control**	**Ethanol**
**Cer C2:0**	0.0	0.0	25.0	0.0	28.6	28.6	33.3	33.3
**Cer C4:0**	0.0	50.0	25.0	33.3	28.6	28.6	33.3	33.3
**Cer C6:0**	0.0	0.0	0.0	0.0	28.6	0.0	0.0	0.0
**Cer C8:0**	0.0	0.0	0.0	33.3	28.6	0.0	0.0	0.0
**Cer C10:0**	40.0	0.0	0.0	0.0	28.6	0.0	33.3	0.0
**Cer C12:0**	40.0	50.0	25.0	66.7	28.6	28.6	33.3	33.3
**Cer C14:0**	200.0	200.0	300.0	400.0	342.9	428.6	300.0	366.7
**Cer C14:1**	40.0	100.0	25.0	33.3	57.1	57.1	100.0	233.3
**Cer C16:0**	40.0	0.0	25.0	33.3	57.1	28.6	33.3	33.3
**Cer C16:1**	40.0	0.0	0.0	33.3	0.0	0.0	0.0	0.0
**Cer C18:0**	40.0	50.0	25.0	0.0	57.1	28.6	33.3	0.0
**Cer C18:1**	120.0	100.0	200.0	233.3	171.4	200.0	166.7	133.3
**Cer C18:2**	0.0	0.0	25.0	33.3	28.6	28.6	33.3	33.3
**Cer C20:0**	40.0	0.0	0.0	0.0	0.0	28.6	0.0	0.0
**Cer C20:1**	0.0	0.0	0.0	0.0	28.6	28.6	33.3	0.0
**Cer C20:2**	40.0	0.0	75.0	33.3	85.7	28.6	33.3	33.3
**Cer C22:0**	120.0	0.0	125.0	133.3	57.1	85.7	0.0	66.7
**Cer C22:1**	360.0	400.0	650.0	1133.3	628.6	628.6	466.7	433.3
**Cer C22:2**	120.0	150.0	200.0	300.0	285.7	285.7	133.3	200.0
**Cer C22:3**	40.0	0.0	75.0	0.0	0.0	57.1	0.0	33.3
**Cer C22:4**	40.0	0.0	0.0	33.3	0.0	0.0	33.3	0.0
**Cer C24:0**	40.0	0.0	0.0	33.3	28.6	28.6	33.3	0.0
**Cer C24:1**	40.0	50.0	50.0	100.0	57.1	85.7	100.0	33.3
**Cer C24:2**	920.0	1000.0	1850.0	2400.0	1400.0	1514.3	1200.0	1100.0
**Cer C24:3**	560.0	700.0	1175.0	1733.3	1342.9	1628.6	700.0	733.3
**Cer C24:4**	80.0	100.0	150.0	133.3	142.9	85.7	133.3	100.0
**Cer C26:0**	280.0	250.0	325.0	400.0	285.7	428.6	266.7	300.0
**Cer C26:1**	40.0	50.0	50.0	33.3	28.6	57.1	33.3	33.3
**Cer C26:2**	0.0	0.0	25.0	0.0	0.0	28.6	0.0	0.0
**Cer C26:3**	0.0	0.0	0.0	33.3	0.0	0.0	0.0	0.0
**Cer C26:4**	40.0	50.0	75.0	66.7	28.6	57.1	33.3	33.3

Supplementary Figure 1: *Full-length blot images for cropped western blots.* A: Figure 1E. B: Figure 3A. C: Figure 3G. D: Figure 4B.

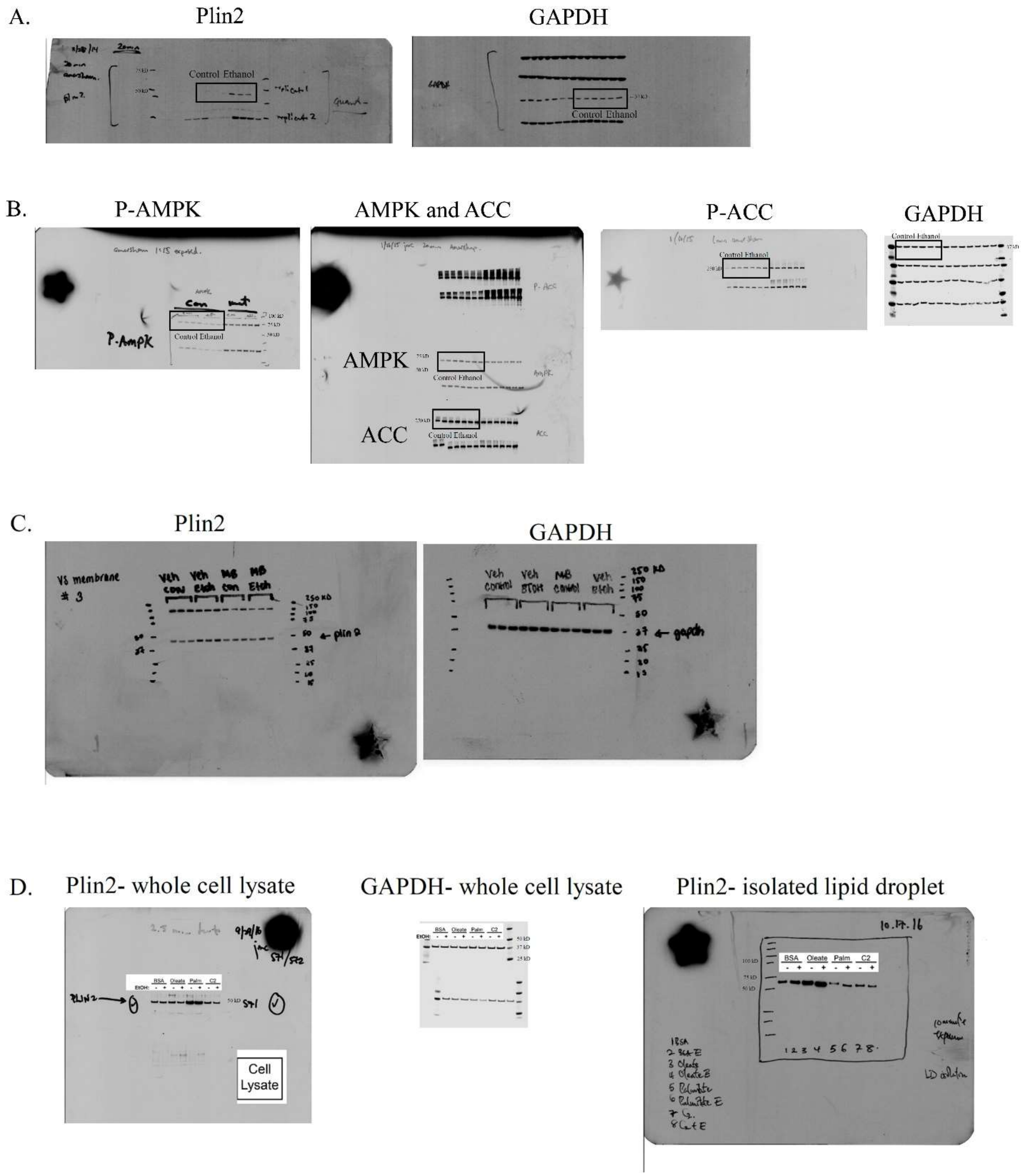

Supplement: Supplementary file 1 — Dataset 1 [file 41598_2018_31025_MOESM1_ESM.pdf]
